# On-Site 4-in-1 Alignment: Visualization and Interactive CAD Model Retrofitting Using UAV, LiDAR’s Point Cloud Data, and Video

**DOI:** 10.3390/s19183908

**Published:** 2019-09-10

**Authors:** Pavan Kumar B. N., Ashok Kumar Patil, Chethana B., Young Ho Chai

**Affiliations:** Graduate School of Advanced Imaging Science, Multimedia, and Film, Chung-Ang University, Seoul 06974, Koreaashokpatil03@hotmail.com (A.K.P.);

**Keywords:** unmanned aerial vehicle, laser scanner, point cloud data, point cloud alignment, retrofitting, cad model, visualization, interactions

## Abstract

Acquisition of 3D point cloud data (PCD) using a laser scanner and aligning it with a video frame is a new approach that is efficient for retrofitting comprehensive objects in heavy pipeline industrial facilities. This work contributes a generic framework for interactive retrofitting in a virtual environment and an unmanned aerial vehicle (UAV)-based sensory setup design to acquire PCD. The framework adopts a 4-in-1 alignment using a point cloud registration algorithm for a pre-processed PCD alignment with the partial PCD, and frame-by-frame registration method for video alignment. This work also proposes a virtual interactive retrofitting framework that uses pre-defined 3D computer-aided design models (CAD) with a customized graphical user interface (GUI) and visualization of a 4-in-1 aligned video scene from a UAV camera in a desktop environment. Trials were carried out using the proposed framework in a real environment at a water treatment facility. A qualitative and quantitative study was conducted to evaluate the performance of the proposed generic framework from participants by adopting the appropriate questionnaire and retrofitting task-oriented experiment. Overall, it was found that the proposed framework could be a solution for interactive 3D CAD model retrofitting on a combination of UAV sensory setup-acquired PCD and real-time video from the camera in heavy industrial facilities.

## 1. Introduction

Due to increase in availability, decreased price, and the development of virtual techniques, 3D scene (Data) acquisition of real-world environments has drawn much interest in related research and development work. The motivation behind these efforts is to represent real-world environments in 3D. These representations have various applications including mapping, renovation, reverse engineering, retrofitting, industrial design, and semantic analysis of complex buildings [1,2].

Various scanners are available due to the increasing acquisition of 3D scenes in different applications. These scanners are based on laser or light-beam return times and can be divided into two groups: high-resolution (dense data) and low-resolution (low data) scanners. High-resolution scanners (e.g., Trimble) need more time to complete a full scan because they generate a highly dense grid of dots. Hence, these are unsuitable for real-time applications.

Meanwhile, low-resolution scanners (Microsoft Kinect V2, Hokuyo and Velodyne LiDAR) are commonly used in real-time applications because they require less time for generating 3D data [3] and are suitable for 3D environment analysis. LiDAR scanners are commonly used in UAVs [4,5], robots [6] and autonomous cars [7]. Hence, laser sensor technology provides accurate geometric information by acquiring the complicated surfaces using various methods.

The sensors used in various applications produce massive amounts of 3D data. The typical interactions with 3D data that include visualization, retrofitting, and presentation are among the biggest challenges at present, because interaction techniques allow users to choose the best possible viewpoint for their analysis.

This work was motivated by the prototype implementation proposed in our previous work [8] and extended to make the system operational in real-time for virtual retrofitting of industrial plants where human reachability is difficult (industrial plants like thermal, petrochemical, and nuclear power stations).

Maintenance and upgrading heavy industrial facilities often need components to be redesigned and/or added. Validating these upgrades (retrofitting) is time-consuming, tedious, and expensive. Hence, virtual retrofitting with 3D models (CAD) is a requirement that can help to make retrofit decisions faster, easier and economical. Therefore, intuitive interaction methodologies for the virtual retrofitting of CAD models are urgently required.

**Figure 1:**
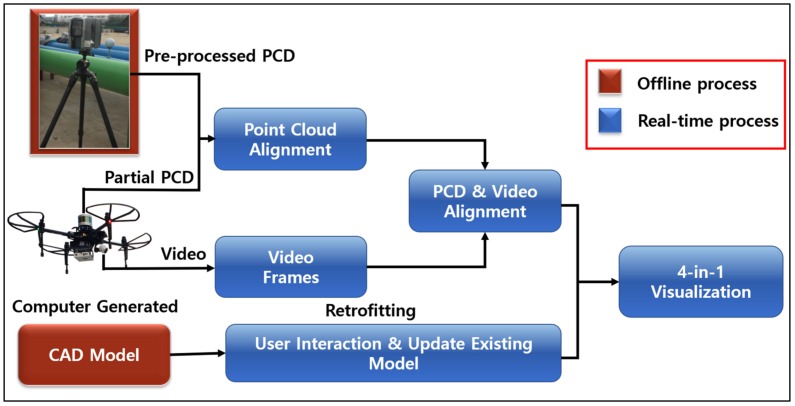
Overall representation of proposed framework.

Figure 1 shows the proposed novel generic framework for virtual retrofitting, intended for making interactive virtual changes and upgrading existing industrial facilities. To align pre-processed PCD with the physical world, partial PCD was acquired from a UAV-mounted LiDAR, and a CAD model was interactively retrofitted in the virtual environment and visualized on a flat monitor.

The rest of this paper is organized as follows. Section 2 discusses earlier work related to point cloud acquisition, visualization, point cloud registration algorithms, and alignment in video. Section 3, Section 4, and Section 5 describe the proposed framework, conduct experiments and obtain results of the proposed framework applied to a practical case study. Finally, this paper’s conclusions and future work are discussed in Section 6.

## 2. Related Works

Adequate visualization facilitates easier, deeper, and better understanding of the data and the underlying physical features for users [9,10,11]. Visualization techniques are considered more intuitive, interactive, and immersive [12]. The interactive manipulation and control of visualization allows users to focus more on their region of interest very quickly [13]. Interactions in a 3D environment give a more realistic symbolic representation of the real-world objects which enhances the users’ memories of the environment [14].

Modern remote sensing technologies such as 3D laser scanners and 3D scene construction based on images are in increasing demand. Laser scanners have been a common tool in the acquisition of 3D object geometry across many industries for applications in civil infrastructure design, maintenance, as-built construction verification, reverse engineering, and part inspection [15,16]. They can acquire 3D shapes with detailed geometrical information. Generally, the 3D data is represented in the form of point cloud models, mesh models and geometric models, among which point cloud models are the basis [17]. Laser scanners measure with accuracies of millimeters to centimeters from the sensor to nearby surfaces at speeds of thousands to hundred thousands of point measurements per second. However, manually acquiring PCD in heavy industrial plants with numerous pipelines is tedious, time-consuming, and laborious [18]. A UAV could provide spatial sensory information at a much higher resolution by inspecting at considerably closer range [19]; it could access many environments in which human access is restricted. UAVs are widely used for various application purposes such as aerial surveillance, environmental monitoring, traffic monitoring, and precision agriculture [20,21] and are capable of carrying payloads over long distances. Thus, a laser scanner mounted on a UAV could map an entire industrial environment and produce a comprehensive PCD.

There has been little work about virtual retrofitting in existing plant facilities in recent decades. In the current framework, we propose virtual retrofitting as opposed to usage of commercial software by professionals [22]. Using CAD models in a retrofitting or upgrade process is much less expensive when a retrofit has been solid-modeled on a computer and analyzed before the part or change is implemented onsite [23]. Therefore, there is a need for CAD model-based retrofitting for a process to re-engineer existing complex facilities.

For the alignment of PCD, the proposed framework uses the Generalized-Iterative Closest Point (G-ICP) registration algorithm [24]. Many efficient registration algorithms have been proposed over the last three to four decades. With regard to accuracy, performance, and robustness, the Iterative Closest Point (ICP) algorithm [25] is best suited for our needs. Several studies have discussed modeling real environments using an RGB-D camera that represents the envirnments as point clouds. The work demonstrated in [26] of 3D mapping and modeling using an RGB-D camera in indoor space investigates recovery from registration failures and coverage through visual inspection. A method proposed in [27] sought to integrate information from video sequences into existing reference 3D point clouds. It shows how to extract local 3D information from the video data, which allows incremental growing, refinement, and update of the existing 3D models.

In our previous work [8], we proposed a novel prototype framework for pipeline virtual retrofitting with 4-in-1 alignment approach, and experiments were conducted by setting an experimental scenario in an indoor environment. Also, our previous work used Velodyne LiDAR for both the PCD acquisition (i.e., pre-processed and partial PCD) as well as for PCD alignment and external GoPro camera were used for the video alignment. The basic setup and preliminary results demonstrated that the 4-in-1 alignment approach can be utilized for the pipeline retrofitting applications.

Hence, the proposed framework utilizes the 4-in-1 alignment approach [8] for an outdoor environment by extending real-time PCD acquisition using a UAV and various sensory setups (Trimble TX5, UAV mounted Velodyne LiDAR and Camera) at an on-site water treatment facility. The proposed current framework provides the precise insertion/modification of the CAD models that are responsible for updating existing industrial facilities in a desktop environment.

## 3. Proposed Retrofitting Framework

This study’s main goal is to develop a framework for virtual retrofitting in a desktop environment. This will provide an approach for upgrading comprehensive engineering models in heavy industrial facilities to support, assist decision-making for, and analyze retrofitting projects. Traditional retrofit projects involve engineers visiting a site multiple times to take field measurements for their designs.

The proposed framework for virtual retrofitting has an impact on reducing errors and interference that is possible in on-site construction works. This section discusses the proposed framework.

### 3.1. 3D Point Cloud Acquisition

The shape of a 3D model with detailed geometry information is acquired during the scanning process, and various techniques are used with a wide range of hardware devices to acquire the 3D model.

#### 3.1.1. Pre-Processed PCD

**Figure 2:**
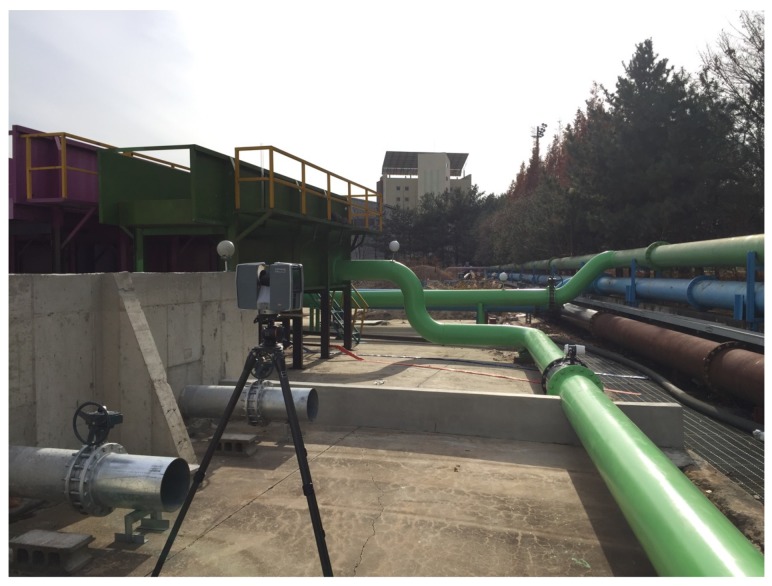
Pre-processed PCD acquisition using a Trimble X5 on-site.

**Figure 3:**
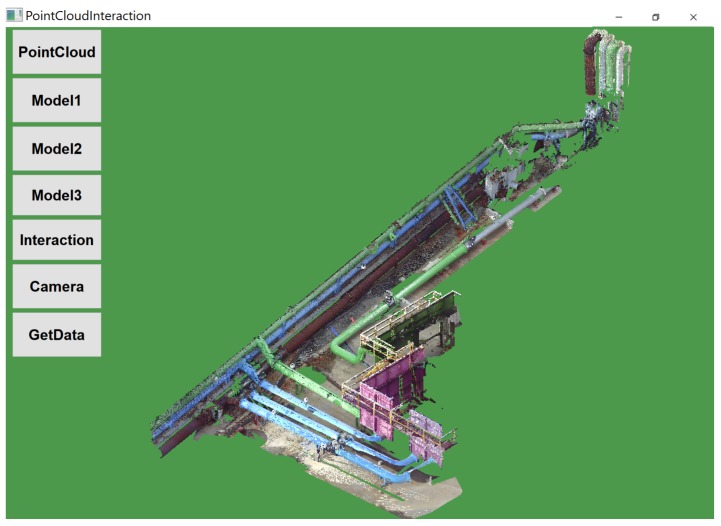
Acquired pre-processed PCD using Trimble X5.

In this study, a water treatment facility at the Korea Institute of Construction Technology was used for the experiment (Figure 2) with various pipe diameters. The commercial Trimble TX5 3D laser scanner, with accuracy of up to ±2 mm, as shown in Figure 2, was used to acquire a pre-processed PCD of the experimental site. A single scan may contain several million 3D points. Since no single scanning position can visualize all surfaces within a facility, scans should be obtained from multiple positions. Hence, the scanner was positioned in different locations. The Trimble TX5 comes with professional software that can register and fuse several scans into a single complete model. Figure 3 shows the resulting pre-processed PCD.

#### 3.1.2. UAV Setup

**Figure 4:**
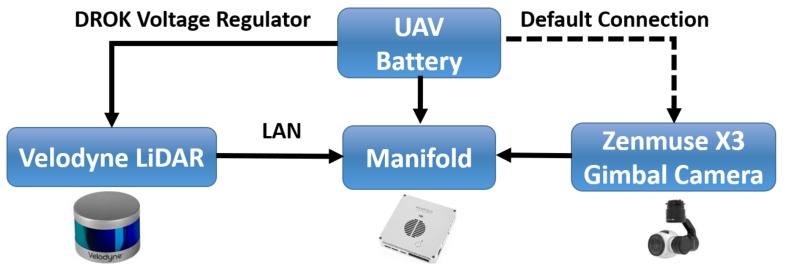
Velodyne sensor, Manifold onboard computer, and Zenmuse X3 hardware integration with UAV.

In this study, a DJI Matrice 100 UAV with TB47D battery was used and the Velodyne LiDAR was mounted on UAV to acquire partial PCD of the scanning environment. The UAV provided stabilized flight and 13 min of hover time with a maximum payload of 1000 g. Table 1 shows the DJI Matrice 100 technical specifications [28]. The UAV was also mounted with a Zenmuse X3 Gimbal camera to get real-time video feed for the video alignment process, as shown in Figure 1. A DROK voltage regulator was used to share the UAV’s battery power with the Velodyne LiDAR. Ethernet cable was used to transfer PCD from Velodyne LiDAR to the Manifold. The whole hardware integration of Velodyne sensor, Zenmuse X3 gimbal camera, and Manifold onboard computer with UAV is shown in Figure 4.

#### 3.1.3. Real-Time Partial PCD

**Figure 5:**
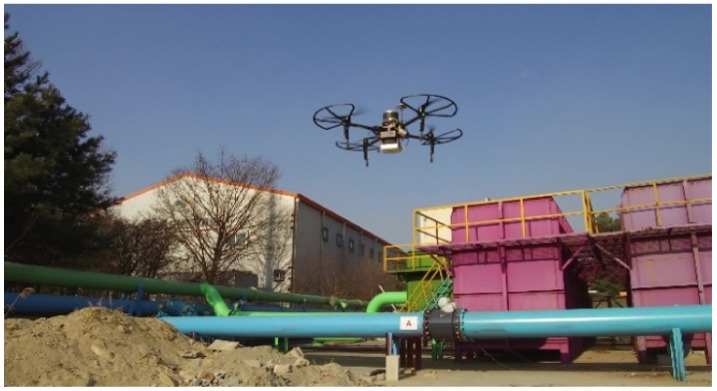
UAV on-site for the partial PCD acquisition using Velodyne Puck LITE.

The Velodyne LiDAR Puck LITE was mounted on a UAV, as shown in Figure 1 and Figure 5, to acquire partial PCD. This is a lightweight version that is specifically designed to meet the relatively low UAV weight restrictions. The sensor is a 16-channel LiDAR scanning 360 in the horizontal and 15 in the vertical field of view.

**Figure 6:**
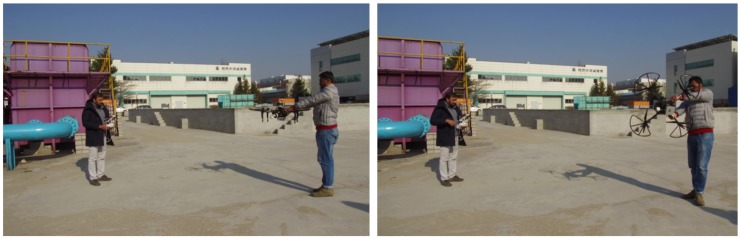
UAV calibration at the experimental site: (**Left**) Holding it horizontally and (**Right**) vertically.

The sensor has low power consumption, scans the environment in 3D at up to 20 Hz while generating about 300,000 points per second with a maximum range 100 m, and weighs 590 g, making it ideal for mounting on a UAV. The orientation of the scanning platform of the UAV at a fixed altitude was obtained through an inertial measurement unit (IMU) sensor (orientation estimation from an IMU sensor is explained in our previous work [5]). Before the acquisition of partial PCD to ensure optimal flight performance, the UAV compass was calibrated with payload (UAV mounted with Velodyne LiDAR, camera, and onboard computer) based on the DJI calibration procedure mentioned in [29] (Figure 6) at the experimental site.

The entire scanning process was implemented in the onboard computer (DJI Manifold) mounted on a UAV using a robot operating system (ROS) framework [30]. This was triggered by a command sent from a remotely connected computer to the onboard computer, and data was transferred to the remote computer via a secure SSH (Secure Shell) for visualization and retrofitting.

### 3.2. PCD Alignment

The very popular iterative closest point [31] algorithm’s variant called G-ICP [24] was used for PCD alignment. It was adopted to check the correct orientation and relative position with pre-processed PCD in a global coordinate system. The alignment in the real world increases the accuracy of the virtual retrofitting. The key features of the G-ICP algorithm are outlined in three steps:

- Determine pairs of corresponding points in pre-processed PCD and partial PCD.
- Estimate a transformation that minimizes the distances between the correspondences.
- Apply the transformation to pre-processed PCD to align with partial PCD.

**Figure 7:**
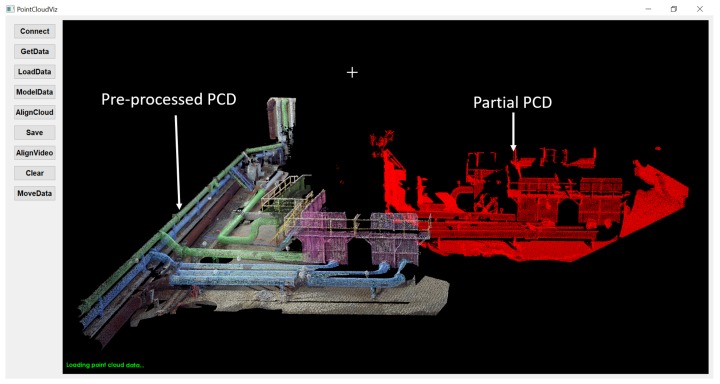
Pre-processed and partial PCD before alignment.

**Figure 8:**
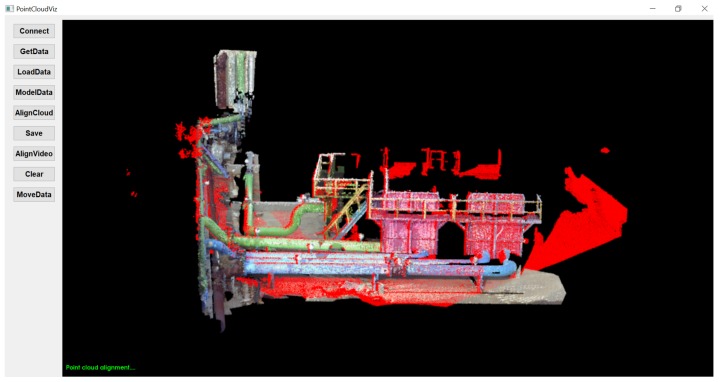
Pre-processed and partial PCD after alignment.

The process of detailed PCD alignment is given in our previous research [8]. Table 2 shows alignment accuracy by implementing G-ICP algorithm. The G-ICP algorithm ran for 16 iterations, where pre-processed PCD transformed to align with physical environment. The transformation between pre-processed and partial PCD occurred in every iteration with change in rotations (roll, pitch, and yaw) and reduced distance from the initial to an aligned position. Figure 7 and Figure 8 show before and after alignment of PCD used in the proposed framework and represent the customized GUI, which provides functionality such as

- Connect—Connects to onboard computer from a remote computer through SSH.
- GetData—Copies the partial PCD from onboard computer to the remote computer.
- LoadData—Visualizes acquired partial PCD from UAV sensory setup.
- ModelData—Loads pre-processed PCD for alignment with partial PCD.
- AlignCloud—Aligns pre-processed PCD with partial PCD using G-ICP algorithm.
- AlignVideo—Aligns pre-processed, partial PCD and retrofitted CAD model with real-time video.

### 3.3. Efficient Visualization and Interactive Retrofitting

The proposed framework provides a means to visualize and analyze a retrofit by interacting with CAD models and PCD for better decision making. The software setup was developed in a C++ programming environment using the visualization toolkit (VTK), an open-source software system [32], and Qt for the GUI.

The VTK pipeline architecture for visualization starts with a source that provides initial data input from files; this is fed as input to the filters, which is optional and helps modify the data in a manner such as conversion, reduction, or merging. Data from the filter were transferred to a mapper, which converts it into tangible objects. In the next step, actors adjust visible properties such as the transparency and color. The remaining work is done by renderers and windows, which create a view-port on the screen where mouse-based interaction could be done. Here, the PCD was rendered for visualization in a desktop environment that permits user analysis and retrofitting by upgrading the existing model. Figure 3 shows the customized GUI application for visualization and provides various interactions functionality such as

- PointCloudData—Loads aligned pre-processed PCD for the visualization.
- Model 1, 2—Enables user to switch between the models for mouse-based interactive retrofitting.
- Interaction—Enables user to perform transformation (translate, rotate, and scale) interactions.
- Camera—Enables to set best view-port for analysis.

The two virtual CAD models were designed and proposed for virtual retrofitting through user interactions. Models were designed by geometrical information acquired utilizing LiDAR.

#### 3.3.1. CAD Model 1

**Figure 9:**
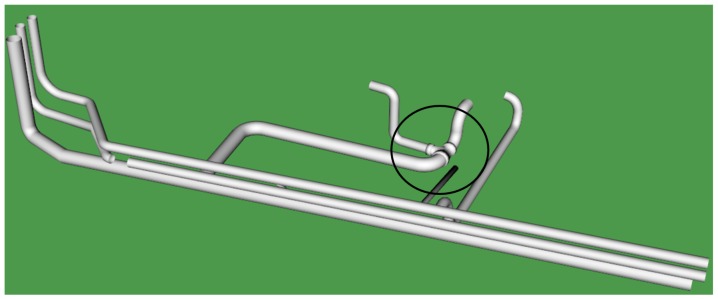
Predefined CAD Model 1.

**Figure 10:**
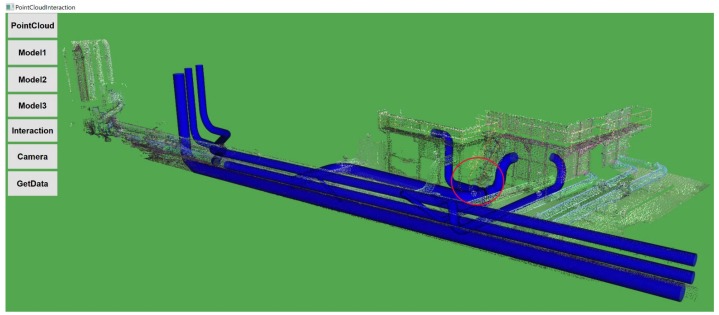
PCD after retrofitting with CAD Model 1.

Figure 9 shows the proposed predefined CAD Model 1 from the Auto-CAD software for virtual retrofitting; this model was designed by keeping the original pipeline facility as a reference. It introduces a T-joint as highlighted in Figure 9 to increase the water flow efficiency. Figure 10 shows the retrofitted Model 1 with PCD by performing mouse-based interactions.

#### 3.3.2. CAD Model 2

**Figure 11:**
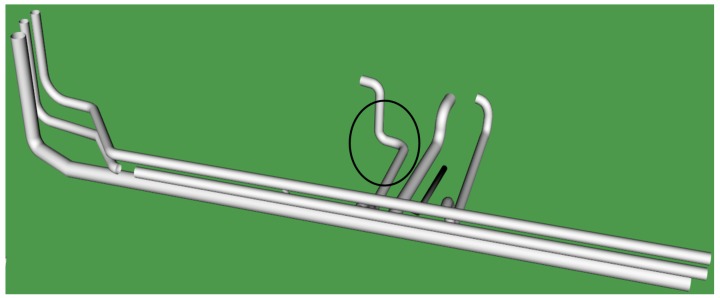
Predefined CAD Model 2.

**Figure 12:**
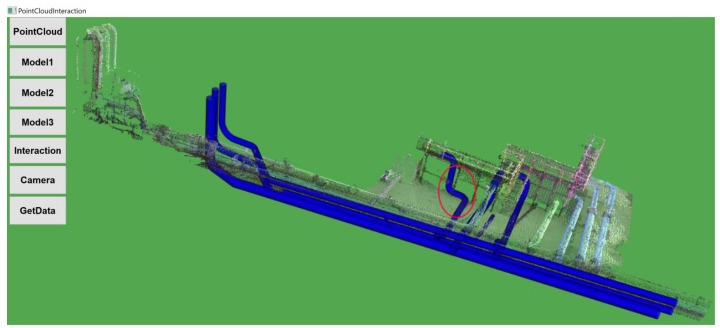
PCD after retrofitting with CAD Model 2.

Figure 11 shows the proposed predefined CAD Model 2, as highlighted in the figure; the original pipeline facility has been replaced by an L-joint that reduces the pipeline complexity and time required for water to flow through the pipeline system. Figure 12 shows Model 2 retrofitted with PCD by performing mouse-based interactions.

### 3.4. 3D Point Cloud Alignment in Video

**Figure 13:**
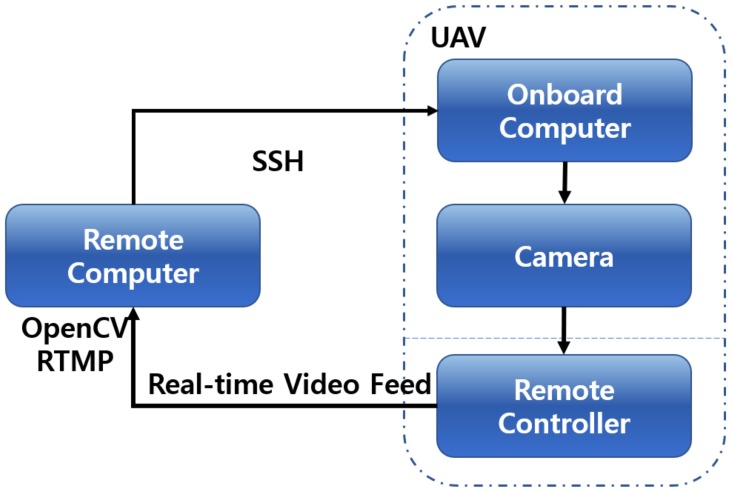
Software setup prepared to get real-time video feed.

A DJI Zenmuse X3 gimbal camera was mounted on a UAV to get the real-time video feed of the real-world environment. Figure 13 shows the setup prepared to get real-time video feed on a remote computer. In our current hardware setup, the onboard computer using ROS had a direct access to the real-time video. Hence, from the remote computer through SSH, video control access was transmitted to the UAV remote controller’s display device (Mobile/Tablet).

The DJI Go application provides the functionality to stream a real-time video from UAV camera to a customized broadcasting channel. Remote computer running on Windows 10 operating system prepared with real-time messaging protocol [33], OpenCV, and open broadcaster software [34] subscribes to that broadcasting channel to get real-time video feed on a remote computer.

Calibration between the sensors (camera and LiDAR sensors) is required to accurately align the PCD information in an image. To calibrate the camera with the LiDAR in the proposed framework, a sensors calibration approach presented in [35] was used. The approach [35] uses a special 3D marker to calibrate and can easily detect correspondence in camera and LiDAR sensors for deterministic estimation of the translation between two sensors. A 3D marker calibration approach enables fine alignment of the sensors before data acquisition from the camera and LiDAR.

**Figure 14:**
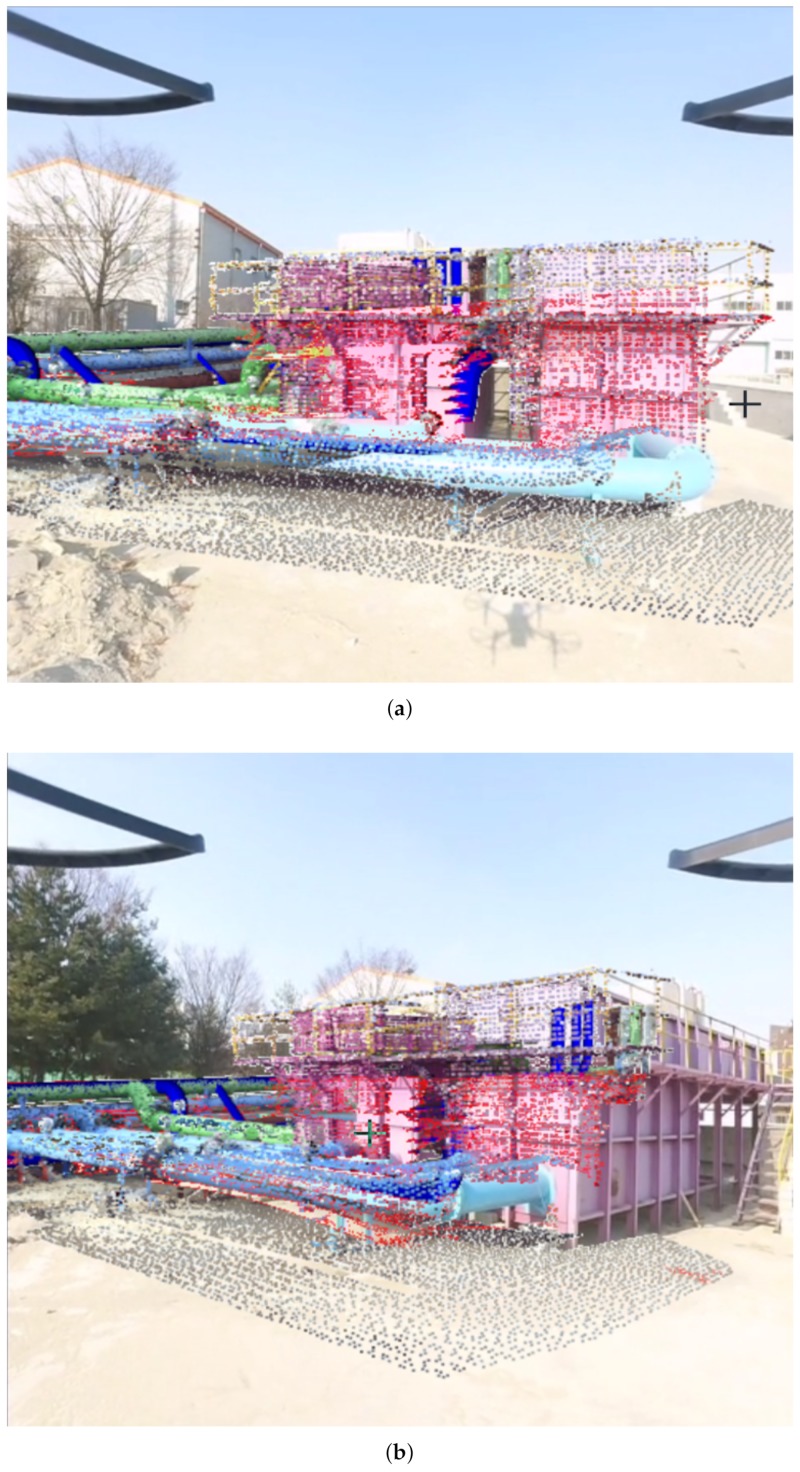
Pre-processed, partial PCD, CAD Model, and Video frame: (**a**) 4-in-1 alignment in first view, (**b**) 4-in-1 alignment in second view.

LiDAR-generated 3D PCD were automatically aligned with the 2D video frames using a frame-to-frame registration method [26]. Before frame-to-frame registration, the PCD in the image plane, the orientation, and the position of the object image plane were estimated as given in our previous research [8]. PCD alignment is a time-consuming process in the 2D image plane; hence, only few frames were periodically selected to register with point cloud. Figure 14a,b shows the results of 4-in-1 alignment in video frames using proposed framework at on-site water treatment facility, with camera views in two different UAV hovering positions. We can recognize aligned pre-processed PCD (RGB) with partial PCD (Red) and retrofitted CAD model (Blue) in a video frame.

## 4. Retrofitting Task Oriented Evaluation

**Figure 15:**
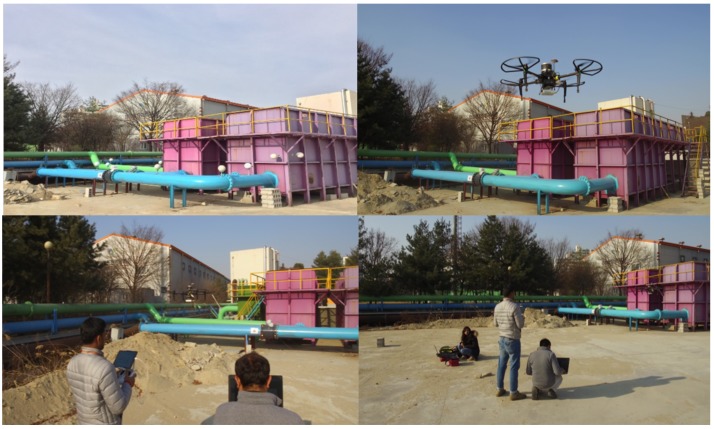
Experimental setup at on-site water treatment facility.

The proposed framework for retrofitting applications was trialed on-site at a water plant facility as shown in Figure 15. Due to technological advancements in the equipment’s, periodically forces to upgrade existing water treatment plants. Retrofitting occurs for many reasons at existing plants such as hydromodifications, reinforcement upgrades, and integration of new technologies.

Presently, most existing retrofitting framework/approach is either traditional which involves physical effort or manually done by a professional with the help of some commercial software. Therefore, the performance of the proposed framework was evaluated based on a retrofitting task and user satisfaction survey by inviting fifteen volunteers. More details about the retrofitting task and participant details are explained in the following subsections.

For the implementation of the proposed framework, development platform includes Alienware laptop running on Windows 10 operating system with Intel (R) CORE (TM) i9-8950 HK, 32 GB random access memory, and an NVIDIA GeForce GTX 1080 GPU (Nvidia Corporation, Santa Clara, CA, USA).

### 4.1. Participants

A group of fifteen participants volunteered to take part (five female and ten male) in the user evaluation study and successfully performed tasks (Tasks 1 and 2). The age of the participants ranged from 25 to 36 years, with a mean (M) of 29.6 years old and standard deviation (SD) = 3.65. All participants were given a brief verbal description of the idea of the evaluation task and they were regular computer users (at least 3 h per day), but none of them had any prior experience with retrofitting.

A separate eight participants were invited for the comparison evaluation task (Task 3). The participants were also regular computer users but with a knowledge of retrofitting.

### 4.2. Retrofitting Task Procedure

Participants took part in the retrofitting task evaluation individually. Prior to starting the task, participants were given a short oral presentation about the user study. It included an introduction to the framework, and instructions on how to use retrofitting GUI with help of interactions such as translation, rotation, and scale. All the participants were required to confirm the understanding of these introductions and the requirements of the experimental task. This was to get the participants familiar with interactions.

After completion of the oral introduction session, participants started the task directly; no earlier training period was provided before the formal task. The task was divided into three categories,

#### 4.2.1. Task 1

In this category of the task, the proposed CAD Model 1, as shown in Figure 9, was placed away either in X- or Y-axis with a little change in orientation from that of PCD. Participants were asked to retrofit CAD Model 1 against the PCD using mouse-based interaction with transformations in five trials as described in Section 3.3 (Interaction functionality).

#### 4.2.2. Task 2

In this category of the task, the proposed CAD Model 2, as shown in Figure 11, was placed away both in X- and Y-axis with more change in orientation from that of PCD. Here, participants need to perform multiple rotation, translation, and scale in order to adjust with the size and orientation of the pipelines in the PCD of the water treatment facility. Participants were asked to perform a retrofitting task in five trials using interaction functionality.

During the above tasks (Section 4.2.1 and Section 4.2.2) for the objective measure, the actual time-to-complete for retrofitting of each model which defines the efficiency based on the accuracy (effectiveness) of completion was recorded for each participant and trial, as shown in Table 3 and Table 4. The very goal of dividing the task was to understand the suitability of the proposed framework for simple and complex retrofitting tasks.

#### 4.2.3. Task 3

To evaluate the performance of the proposed framework, a comparison evaluation was carried out in repeated measures by considering control and experimental group. Here, we invited eight separate participants (Section 4.1), assigned to be the control group, and the other eight participants among the fifteen were considered as an experimental group. The goal of the experiment was the same as described for Task 2 (Section 4.2.2), and participants in the experimental group used our proposed framework to perform retrofitting task. On the other hand, participants in the control group used open-source software CloudCompare [36] to perform the same task. The independent variable was the retrofitting interface, by which we compared the proposed framework’s performance (time-to-complete) with CloudCompare.

Once the retrofitting tasks (Section 4.2.1, Section 4.2.2 and Section 4.2.3) have been finished, we collected the participants’ qualitative feedback on the proposed framework by using questionnaire and short interview for the subjective measure.

## 5. Results

In this section, we report the results of the participants’ tasks evaluation through objective and subjective measures.

### 5.1. Objective Measures for Task 1 and Task 2

#### 5.1.1. Efficiency

**Figure 16:**
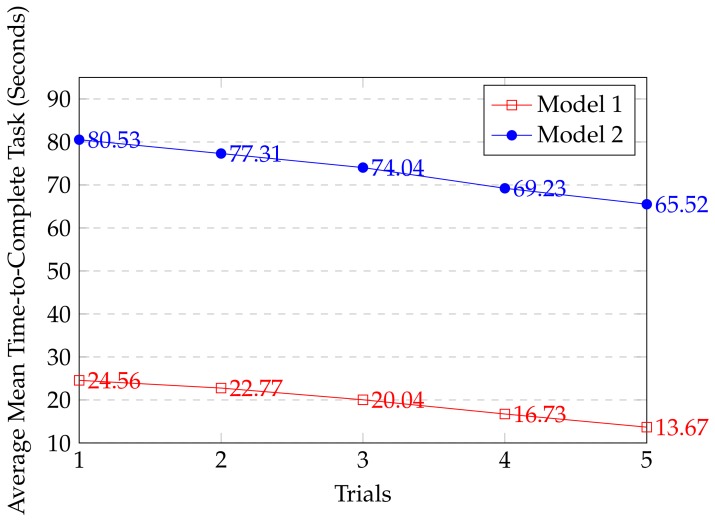
Average mean time-to-complete the tasks for each model and trial by participants.

Efficiency was defined as time-to-complete both the retrofitting task and used as one of the objective measures in the evaluation of the proposed framework. Overall, these tasks were performed by each participant in five trials, providing a total of 150 experiments for both CAD models. Table 3 and Table 4 show the overall mean time-to-complete retrofitting tasks five trials for each model. For Model 1, we can clearly see that the M and SD for trial 1 ($M_{T1}$ = 24.56, $SD_{T1}$ = 3.24 ) to trial 5 ( $M_{T5}$ = 13.68, $SD_{T5}$ = 2.36) on average significantly time decreased by 20 % for each trials. Similarly, for Model 2 the M and SD for trial 1 ( $M_{T1}$ = 80.53, $SD_{T1}$ = 4.93 ) to trial 5 ( $M_{T5}$ = 65.52, $SD_{T5}$ = 4.32) on average significantly time decreased by 20 % for each trial. Figure 16 shows the mean time-to-complete each retrofitting task in five trials. It shows that the participants become more acquainted with the system in every trial.

#### 5.1.2. Accuracy

In addition to efficiency, accuracy was measured as a second objective measure in the evaluation of proposed framework. For accuracy evaluation, we considered:

- Four key points in each CAD model and PCD.
- For each user intended interaction, distance between key points in CAD model and PCD was checked against minimum threshold distance ($TD_{min}$)(in this paper $TD_{min}$ set to 2 mm).

Participants were allowed to carry out the retrofitting task by interactions till CAD model color turned to blue when CAD model transformed within the $TD_{min}$. The color change in the CAD model represented the successful retrofit and end of the retrofitting task.

### 5.2. Objective Measures for Task 3

**Figure 17:**
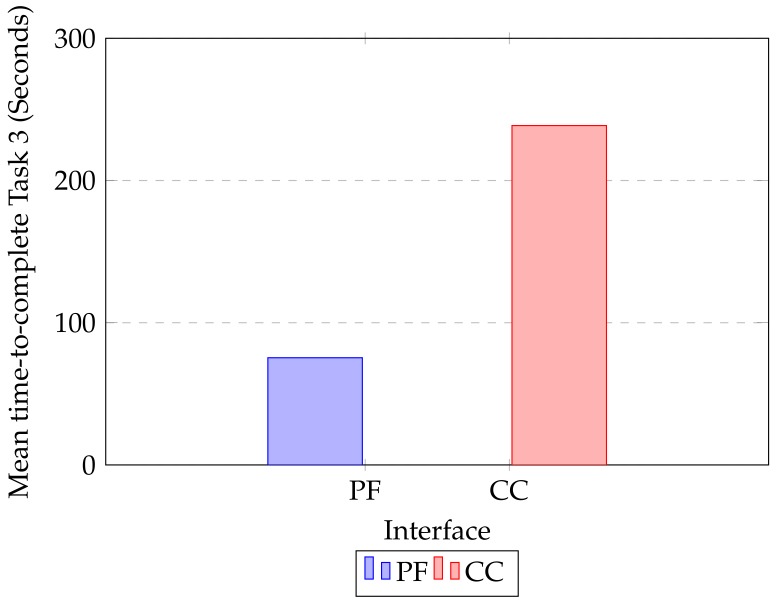
Mean time-to-complete Task 3 using Proposed Framework (PF) and CloudCompare (CC).

For the objective measure of the proposed framework, we observed the performance of the interface between two groups (control and experimental) in Task 3. Table 5 shows the control group participant’s time-to-complete retrofitting Model 2 using CloudCompare interface. On the other hand, we considered Table 4’s first eight participant’s time-to-complete for comparison against CloudCompare. A Paired T-test showed a highly significant difference in time-to-complete retrofitting tasks between the proposed framework and CloudCompare, T(15) = 2.78, p = 4.35 ×$10^{-6}$. Figure 17 shows the overall mean time-to-complete for each retrofitting process, and we can clearly see that using our proposed framework interface ($M_{PF}$ = 75.38, $SD_{PF}$ = 0.36) in the experiment participants take significantly less time on average than when using CloudCompare ($M_{CC}$ = 238.63, $SD_{CC}$ = 1.97).

### 5.3. Subjective Measure Results for Task 3: User Satisfaction and Feedback from Questionnaire

To determine the user satisfaction for the proposed framework a user satisfaction appropriate questionnaire [37,38] were adopted and filled by the participants after the end of the task. These are simple and widely used survey questions developed for a subjective measure of system usability.

All the questions, five in total were designed in favor of immersion, level of consistency, level of efficiency, interface quality, and ease of use to perform retrofitting. Participants were asked to rate their usability responses with a 5-point scale (rating from 1 (strongly disagree) to 5 (strongly agree)).

- Q1: How well could you interact or manipulate models in the virtual environment?
- Q2: How well could you investigate models from multiple viewpoints?
- Q3: How helpful was the interface when performing the assigned tasks?
- Q4: I felt very confident using the system.
- Q5: I needed to learn a lot of things before using it.

**Figure 18:**
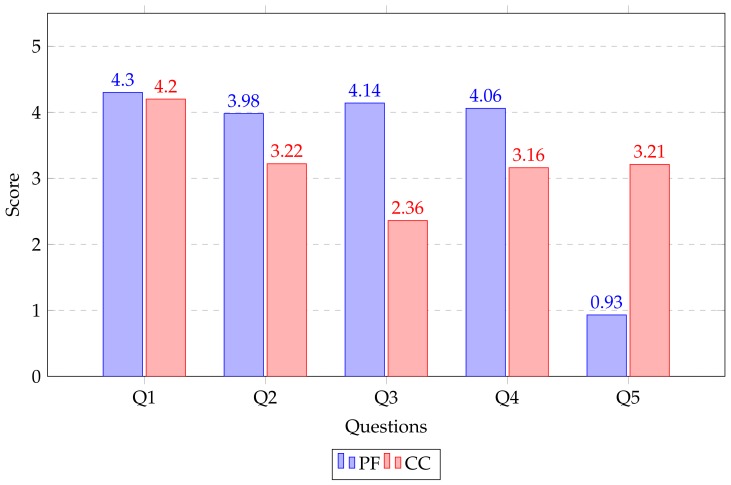
The average ratings of the (5 point scale: 1 - strongly disagree, 2 - disagree, 3 - neutral, 4 - agree, 5 - strongly agree) user satisfaction questionnaire for Proposed Framework (PF) and CloudCompare (CC).

Figure 18 shows a representation of the average ratings of the questionnaire. Almost all ratings lie between neutral and strongly agree, which means that there are no negative impressions about the proposed framework. However there is no significant difference in ratings of Q1; in fact the mean rating of proposed framework ($M_{Q1PF}$ = 4.30, $SD_{Q1PF}$ = 0.50) is still slightly better than CloudCompare ($M_{Q1CC}$ = 4.20, $SD_{Q1CC}$ = 0.37) regarding the user feedback on interaction/manipulation of models in the virtual environment. Question Q5 obtained a minimum rating for the proposed framework. It shows that participants need not learn more things before engaging in retrofitting tasks as compared to CloudCompare. Since the proposed framework interface provides more intuitive interactions and simple functionality.

From the short interview conducted at the end of the study, participants were asked to state an overall preference of the proposed framework. A majority of the participants felt that the proposed framework will be very useful in industrial facilities because it reduces the labors cost, time and manual effort. It is also very efficient in order to perform modification and upgrading of an existing facility.

### 5.4. Further Discussion on Observation Results and Limitation

From the results of the objective measures, it is clearly indicated that over the trials users will get acquainted with the framework within less time over each trail. This tells us that the user can intuitively retrofit predefined CAD models and analyze the retrofitted industrial facilities to make decisions before actual implementation.

Moreover, the user assessment was consistent with the objective results. On the other hand, during tasks, participants noticed that there was a delay in response during interaction and visualizing PCD. This is due to the large size of PCD; we will consider this limitation as a future scope to introduce down-sampling methods to increase the response time.

## 6. Conclusion and Future work

Maintenance and upgrading plant facilities often need components to be redesigned and/or added. Validating these upgrades (retrofitting) is time-consuming, tedious, and expensive. Hence, virtual retrofitting with 3D models (CAD) is a requirement that can help to make retrofit decisions faster, easier, and more economical. This paper proposes a novel generic framework for an interactive 3D CAD model based retrofitting and an efficient 4-in-1 alignment in a desktop environment before physical on-site implementation.

An on-site trial was carried out using the proposed framework at a water treatment facility in order to evaluate efficiency, ease of use and performance. Also, task-oriented evaluation in terms of objective measure and a user satisfaction questionnaire were done to understand the subjective measures with 15 participants over trials. The overall results inferred that the proposed framework could be a solution to perform virtual interactive retrofitting by reducing labors cost, time, and manual effort before actual on-site upgrading.

The future work direction involves investigation on comparison, and more usability studies. Also, the implementation of immersive visualization in the head-mounted display with interaction methods for more understanding of the actual environment scene should be done. In the proposed framework, the UAV was hovering at stable height during the trial. Future work can also include implementation of autonomous path-planning to acquire the PCD from different views and incorporation of GNNS for better position accuracy. Further, we also intend to improve the alignment of video with LiDAR data by considering calibration between LiDAR and camera.

## Figures and Tables

**Table 1:** Technical specifications for the DJI Matrice 100.

Parameters	Values
UAV type	Quadcopter, with customizable and programmable platform
Battery	Intelligent flight battery 5700 mAh LiPo 6S
Video output	USB, High-Definition Multimedia Interface-Mini
Maximum speed of	Ascent: 5 m/s, Descent: 4 m/s
Operating temperature	-10C to 40C

**Table 2:** Point cloud alignment accuracy using G-ICP algorithm.

Iterations		Alignment			
		Distance (mm)	Roll (°)	Pitch (°)	Yaw (°)
16	1	15.48	−3.5	−1.9	8.7
	8	7.83	−2.4	−1.15	6.6
	16	3.55	−1.3	−1.3	3.8

**Table 3:** Task 1: Participants’ time taken for retrofitting Model 1.

Trials	Time Taken for Retrofitting in Seconds Model 1														
	P1	P2	P3	P4	P5	P6	P7	P8	P9	P10	P11	P12	P13	P14	P15
1	24.01	22.12	30.78	21.74	26.10	23.85	32.18	24.86	26.70	23.86	23.71	24.06	22.60	21.71	20.12
2	21.58	20.75	23.84	22.81	23.71	21.93	23.02	24.10	26.08	25.10	21.30	20.90	21.76	22.90	21.70
3	17.73	21.65	25.84	18.46	24.75	21.48	21.41	19.71	21.81	19.78	18.49	17.61	16.08	17.43	18.39
4	13.68	14.56	20.86	17.32	19.40	18.74	17.19	17.84	19.53	16.40	15.70	15.08	13.80	15.94	14.97
5	10.86	12.27	17.25	14.92	16.74	15.08	15.71	15.60	16.46	12.08	13.08	11.08	11.00	12.00	10.98

**Table 4:** Task 2: Participants’ time taken for retrofitting Model 2.

Trials	Time Taken for Retrofitting in Seconds Model 2														
	P1	P2	P3	P4	P5	P6	P7	P8	P9	P10	P11	P12	P13	P14	P15
1	78.17	82.00	87.10	76.01	81.50	79.49	88.89	83.47	74.17	72.14	85.60	84.71	81.64	77.10	75.92
2	72.06	78.96	81.90	75.08	80.84	78.97	85.15	81.77	70.34	67.93	80.17	81.97	77.41	73.50	73.64
3	75.74	74.10	79.71	71.47	78.47	76.60	83.94	77.51	68.98	62.97	73.41	76.18	72.61	69.60	69.36
4	63.18	70.18	73.40	66.85	72.14	70.16	78.97	73.90	65.35	62.40	68.73	72.96	69.47	65.19	65.49
5	60.06	63.48	71.84	64.52	69.60	67.47	71.31	69.59	61.49	59.01	64.73	70.84	65.10	61.52	62.17

**Table 5:** Task 3: Control group participant’s time taken for retrofitting Model 2.

Trials	Time Taken for Retrofitting in Seconds Model 2							
	P1	P2	P3	P4	P5	P6	P7	P8
1	240.36	235.80	270.84	284.52	265.50	246.81	262.81	290.53
2	215.85	215.70	250.15	255.85	260.83	225.63	268.75	275.62
3	206.70	200.48	235.19	250.45	262.51	220.81	254.36	263.55
4	195.08	195.62	230.18	234.58	255.48	215.76	252.84	245.81
5	190.50	190.48	215.38	229.54	235.64	210.61	245.73	242.61

## Abbreviations

The following abbreviations are used in this manuscript:

UAV	Unmanned aerial vehicle
PCD	Point cloud data
CAD	Computer-aided design
G-ICP	Generalized-iterative closest point
GUI	Graphical user interface
VTK	Visualization toolkit
